# Real-time correction of gain nonlinearity in electrostatic actuation for whole-angle micro-shell resonator gyroscope

**DOI:** 10.1038/s41378-024-00818-x

**Published:** 2024-11-05

**Authors:** Sheng Yu, Jiangkun Sun, Yongmeng Zhang, Xiang Xi, Kun Lu, Yan Shi, Dingbang Xiao, Xuezhong Wu

**Affiliations:** National University of Defense Technology, Changsha, 410073 China

**Keywords:** Electrical and electronic engineering, Physics

## Abstract

MEMS gyroscopes are well known for their outstanding advantages in Cost Size Weight and Power (CSWaP), which have inspired great research attention in recent years. A higher signal-to-noise ratio (SNR) for MEMS gyroscopes operating at larger vibrating amplitudes provides improved measuring resolution and ARW performance. However, the increment of amplitude causes strong nonlinear effects of MEMS gyroscopes due to their micron size, which has negative influences on the performance. This paper carries out detailed research on a general nonlinear mechanism on the sensors using parallel-plate capacitive transducers, which is called the gain nonlinearity in electrostatic actuation. The theoretical model established in this paper demonstrates the actuation gain nonlinearity causes the control-force coupling and brings extra angle-dependent bias with the 4^th^ component for the whole-angle gyroscopes, which are verified by the experiments carried out on a micro-shell resonator gyroscope (MSRG). Furthermore, a real-time correction method is proposed to restore a linear response of the electrostatic actuation, which is realized by the gain modification with an online parameter estimation based on the harmonic-component relationship of capacitive detection. This real-time correction method could reduce the 4^th^ component of the angle-dependent bias by over 95% from 0.003°/s to less than 0.0001°/s even under different temperatures. After the correction of actuation gain nonlinearity, the bias instability (BI) of whole-angle MSRG is improved by about 3.5 times from 0.101°/h to 0.029°/h and the scale factor nonlinearity (SFN) is reduced by almost one order of magnitude from 2.02 ppm to 0.21 ppm.

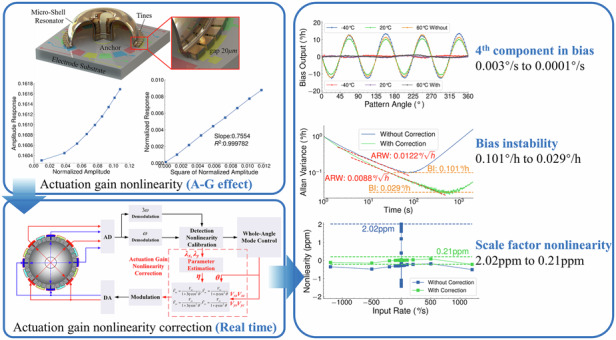

## Introduction

Gyroscopes, as inertial sensors, are the key devices in many systems, such as Gyrocompass, Inertial Measurement Units, Inertial Navigation Systems, and Attitude Heading Reference Systems^1^. Compared with Mechanical Gyroscopes, Ring Laser Gyroscopes, and Fiber-Optic Gyroscopes, Micro-electromechanical system (MEMS) vibratory gyroscopes show their advantages in cost, size, weight, power, and integration^1–3^, which have inspired great research attention over the last two decades.

MEMS gyroscopes are always operated at larger vibrating amplitude to achieve a higher signal-to-noise ratio (SNR) and lower Angle Random Walk (ARW)^4^. However, due to their micron scale, nonlinear mechanisms and phenomena are always observed in MEMS resonators when the vibrating amplitude increases, for example, the geometrical and electrostatic nonlinearity in disk resonator gyroscope^5^, cylindrical resonator gyroscope^6^ and quad-mass gyroscope^7^, the nonlinear couplings and cubic rigidity nonlinearity in ring resonator gyroscope^8^, the electro-mechanical nonlinearities and nonlinear capacitive sensing in Dual-Foucault-Pendulum (DFP) gyroscope^9^, the nonlinear anisotropic damping in annular disc vibratory gyroscope^10^ and the capacitive displacement detection nonlinearity in tuning fork and micro-shell resonator gyroscope^11,12^.

The nonlinear effects of vibratory gyroscopes have attracted extensive attention of researchers in recent years. Pavel presented a characterization method for the coefficients of conservative and dissipative nonlinearities in MEMS resonators by the ring-down response^13^. Maslov constructed the mathematical models of cylindrical resonators taking into account the nonlinearities caused by electrostatic excitation, which shows that cubic nonlinearity leads to gyroscope drifts and quadratic nonlinearity has influences on the amplitude control^14^. Sarah addressed the impact of the Duffing nonlinearity on the disk resonator gyroscope operating in the whole-angle mode, which results in an angle-dependent frequency split and causes negative influences on the bias and scale factor^15^. Zhongxu Hu discovered the nonlinear effects from capacitive transducers of the ring resonator gyroscope would cause the 4^th^ harmonic component of the precession rate, resonant frequency, and quadrature control, which limits the performance of the whole-angle gyroscope^16^. Chouvion found that electrostatic nonlinearity in the ring resonator gyroscope would introduce coupling between the modes which reduces the sensor performance under severe shock^17^. Vatanparvar demonstrated that the electrostatic nonlinearity of DFP gyroscopes would cause noise degradation and scale-factor instability in the case of open-loop operation modality^18^. Thus, increasing the amplitude to achieve a higher SNR will also bring negative influences of strong nonlinear effects on the performance of gyroscopes.

Considering the negative influences of nonlinear effects, a reduction of the vibrating amplitude for the DFP gyroscope can directly weaken the Duffing and quintic nonlinearity and instead improve the ARW and Bias Instability (BI) by about 3 times^19^. Furthermore, there are also some novel methods for correction of the nonlinear terms to restore the linear response or compensation of the nonlinear effects to eliminate the negative influences. Qingsong Li has reported a novel method to reduce the electrostatic and capacitive nonlinearities in a disk resonator gyroscope based on the vibration amplification effect by using the inner electrodes for actuation and the outer electrodes for detection^20^. The angle-dependent frequency mismatch from the Duffing nonlinearity can be compensated by applying a tuning voltage which varies as a function of pattern angle^15^. The amplitude-frequency coupling caused by Duffing nonlinearity can be compensated using a nonlinear feedback loop^21^. The nonlinearity of capacitive displacement detection can be calibrated by removing the extra nonlinear term based on the harmonic component relationship^12^ or parameter estimation^16^. By these methods, the amplitude of the gyroscope does not need to be reduced to make a compromise between better SNR performance and negative influences of nonlinear effects.

Apart from above nonlinear effects, there is another type of nonlinearity in vibratory gyroscopes that has received less attention, which is a nonlinear response between the applied voltage and the acquired force when utilizing electrostatic actuation, resulting in the nonlinearity of actuation gain. A mathematical model of a hemispherical resonator gyroscope (HRG) with flat electrodes shows a nonlinear actuation gain which grows with the increment of vibrating amplitude^22^. However, their influences on the gyroscope and relevant correction approaches have not been studied. A method for electrostatic force linearization is presented by forming the reference voltage as a function of the estimated vibration displacement^23^. However, there is just a theoretical deduction without any experimental verification.

This paper conducts detailed research on the gain nonlinearity in electrostatic actuation about the influence mechanism and suppression approach for the micro-shell resonator gyroscope (MSRG) working on the whole-angle mode. Compared with the force-to-rebalance (FTR) mode, the whole-angle mode provides the vibratory gyroscope with unlimited bandwidth and measurement range, stable scale factor, and precise angle output^24–27^. And the micro-shell resonator has the potential to achieve an ultrahigh *Q* factor of over 4.5 million and excellent structural symmetry with a frequency mismatch of 32 mHz^28,29^. Therefore, combining a micro-shell resonator and whole-angle mode is expected to achieve high-performance MEMS gyroscopes, and further improvements can be achieved by eliminating the nonlinearity of actuation gain under large vibrating amplitude.

This paper builds the model of gain nonlinearity in electrostatic actuation and combine it with Lynch’s theory^30^ of whole-angle mode to derive dynamical control equations of the gyroscope operating in the nonlinear regime. The simulations and experiments demonstrate that the actuation gain nonlinearity causes the force coupling between control loops and brings extra angle-dependent bias with the 4th component. The normalized amplitude is used as the correction parameter to modify the actuation gain to the linear value, which can be estimated in real time based on the relationship between the first and third harmonic components in capacitive detection signals. With the real-time correction of the actuation gain nonlinearity, the control loops are decoupled and the 4th component in the angle-dependent bias is removed, even at different temperatures. Therefore, for whole-angle MSRG, such a real-time correction method can reduce the bias of the device output and improve the performance of both bias instability (BI) and scale factor nonlinearity (SFN), especially when operating at large amplitudes for a better noise level.

## Results

### Device structure and model of gain nonlinearity in electrostatic actuation

**Fig. 1:**
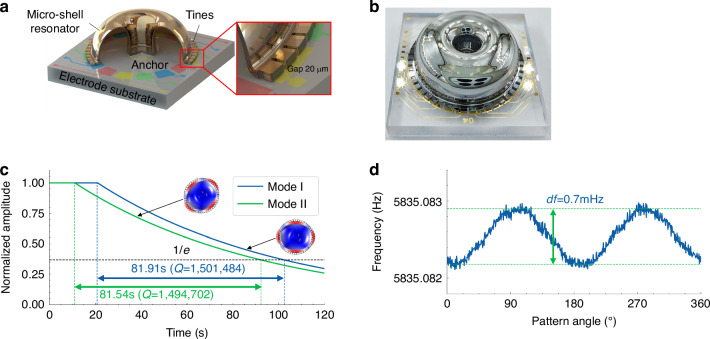
The structure and characteristics of the micro-shell resonator. **a** Structural design of the micro-shell resonator. **b** Photo of real device of the micro-shell resonator. **c** Ring-down tests of two *n* = 2 wineglass modes. **d** Frequency mismatch of the resonator

The structure of the micro-shell resonator is shown in Fig. 1a, which consists of a micro-shell bonded to a planar electrode substrate through the anchor by adhesive. Both the shell and substrate are made of fused silica, which provides the resonator with stable resonance properties within the whole temperature range. The interior surface of micro shell is coated by chromium (Cr) and gold (Au), and the rectangular shape tines around the perimeter collaborate with the electrodes in the substrate to form planar capacitances^31^. The geometry dimensions of the structure are designed to have a micro shell with 4 mm height and 12 mm diameter, as well as a capacitance gap of 20*μ*m. The photo of the real device of the micro-shell resonator is shown in Fig. 1b. Two *n* = 2 wineglass modes of the micro-shell resonator are chosen as working modes, which can achieve long decay times of over 81.5 s and high *Q*-factors of about 1.5 M, as shown in Fig. 1b. And the frequency mismatch of the micro-shell resonator can reach less than 1mHz with the electrostatic tuning, as shown in Fig. 1c.

**Fig. 2:**
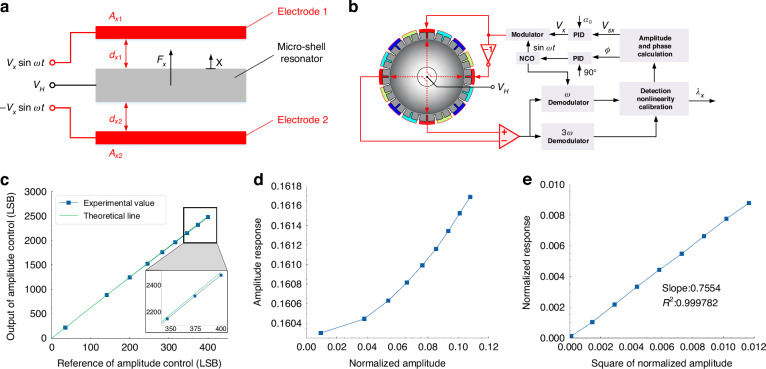
The gain nonlinearity in electrostatic actuation of the micro-shell resonator. **a** Schematic diagram of differential electrostatic actuation. **b** Block diagram of the micro-shell resonator controlled to resonate with the calibration of the detection nonlinearity. **c** The output of the amplitude control loop under different reference values. **d** The amplitude responses of the micro-shell resonator between the input exciting voltages and the output detecting vibration. **e** The normalized responses versus the square of the normalized amplitudes

The micro-shell resonator is excited to vibrate by the electrostatic actuation of the planar capacitances. When applying the differential exciting voltage as ±*V*_*x*_sin*ωt* to the differential actuation electrodes in the substrate as shown in Fig. 2a, the electrostatic force in the micro-shell resonator can be expressed as

1 $$|{F}_{x}|=\frac{\varepsilon {A}_{x1}}{2{({d}_{x1}-x)}^{2}}{({V}_{H}+{V}_{x}\sin \omega t)}^{2}-\frac{\varepsilon {A}_{x2}}{2{({d}_{x2}+x)}^{2}}{({V}_{H}-{V}_{x}\sin \omega t)}^{2}$$

where *x* represents the vibration displacement of the resonator, *V*_*H*_ is the DC bias voltage applied in the resonator, *d*_*x*1_, *d*_*x*2_, *A*_*x*1_, and *A*_*x*2_ represent the gaps and areas of planar capacitors, and *ε* is the dielectric constant. After expanding in power series and neglecting the higher terms above 3^rd^ order, the resonant part in the electrostatic force can be calculated as

2 $$|{F}_{x}|=\left[\left(\frac{\varepsilon {A}_{x1}}{{d}_{x1}^{2}}+\frac{\varepsilon {A}_{x2}}{{d}_{x2}^{2}}\right)+3\left(\frac{\varepsilon {A}_{x1}}{{d}_{x1}^{4}}+\frac{\varepsilon {A}_{x2}}{{d}_{x1}^{4}}\right){x}^{2}\right]{V}_{H}{V}_{x}\,\sin \omega t+\left(\frac{\varepsilon {A}_{x1}}{{d}_{x1}^{3}}+\frac{\varepsilon {A}_{x2}}{{d}_{x2}^{3}}\right){V}_{H}^{2}x+2\left(\frac{\varepsilon {A}_{x1}}{{d}_{x1}^{5}}+\frac{\varepsilon {A}_{x2}}{{d}_{x2}^{5}}\right){V}_{H}^{2}{x}^{3}$$

The second term represents the electrostatic negative stiffness which can be eliminated by the mode matching method^32^ and the third term represents Duffing nonlinearity which can also be compensated by angle-dependent tuning voltage^15^. The first term can be rewritten as

3 $$|{F}_{x}|={k}_{dx}\left[1+3{\left(\frac{x}{{d}_{x0}}\right)}^{2}\right]{V}_{x}\,\sin \omega t$$

where

$${k}_{dx}=\left(\frac{\varepsilon {A}_{x1}}{{d}_{x1}^{2}}+\frac{\varepsilon {A}_{x2}}{{d}_{x2}^{2}}\right){V}_{H},{d}_{x0}^{2}=\left(\frac{{A}_{x1}}{{d}_{x1}^{2}}+\frac{{A}_{x2}}{{d}_{x2}^{2}}\right)/\left(\frac{{A}_{x1}}{{d}_{x1}^{4}}+\frac{{A}_{x2}}{{d}_{x1}^{4}}\right)$$

*k*_*dx*_ represents the linear actuation gain and *d*_*x*0_ represents the equivalent gap. Considering the closed control for phase locking at 90° and amplitude maintaining at *a*_0_, as shown in Fig. 2(b), the effective force can be expressed as

4 $${f}_{x}={k}_{dx}\left[1+3{\left(\frac{{a}_{0}\cos \omega t}{{d}_{x0}}\right)}^{2}\right]{V}_{x}\,\sin \omega t\approx {k}_{dx}\left[1+\frac{3}{4}{\left(\frac{{a}_{0}}{{d}_{x0}}\right)}^{2}\right]{V}_{x}\,\sin \omega t$$

There is a nonlinear actuation gain instead of a constant value, which has an extra item proportional to the square of the vibrating amplitude, as explained in Eq. (4). With the calibration of capacitive displacement detection nonlinearity^12^, the output exciting voltage from the closed loop can be expressed as

5 $${V}_{x}=\frac{2\omega {V}_{sx}}{{k}_{sx}{k}_{dx}\left[1+\frac{3}{4}{\left(\frac{{V}_{sx}}{{k}_{sx}{d}_{x0}}\right)}^{2}\right]{\tau }_{x}}$$

where *V*_*sx*_ is the detected amplitude of the vibrating signal under the calibration of capacitive displacement detection nonlinearity and *k*_*sx*_ is the linear detection gain. Therefore, the response can be obtained as

6 $${G}_{x}=\frac{{V}_{sx}}{{V}_{x}}={k}_{sx}{k}_{dx}\frac{{\tau }_{x}}{2\omega }\left(1+\frac{3}{4}{{\lambda }_{x}}^{2}\right),\frac{{G}_{x}-{G}_{0}}{{G}_{0}}=\frac{3}{4}{{\lambda }_{x}}^{2}$$

where *λ*_*x*_ represents the normalized amplitude, which can be estimated from the harmonic-component relationship in capacitive detection during the process of detection nonlinearity calibration^12^ as

7 $$A={x|}_{\cos \omega t}={k}_{sx}\left(1+\frac{3}{4}{{\lambda }_{x}}^{2}\right)|x|,B={x|}_{\cos 3\omega t}={k}_{sx}\frac{1}{4}{{\lambda }_{x}}^{2}|x|,{{\lambda }_{x}}^{2}=\frac{4B}{A-3B}$$

where *A* and *B* are the amplitudes of the first and third harmonic components of the detected vibrating signal, respectively.

To verify the model of gain nonlinearity in electrostatic actuation, a micro-shell resonator is controlled to resonate at different vibrating amplitudes, as shown in Fig. 2b. The demodulated components are calibrated for capacitive displacement detection nonlinearity before the controls of phase locking and amplitude maintenance. The different reference values of the amplitude-controlling loop and the corresponding applied exciting voltages are recorded. Meanwhile, the normalized amplitude *λ*_*x*_ at every point is calculated during the process of detection nonlinearity calibration based on Eq. (7). According to Eqs. (5)–(6), the applied exciting voltages growth nonlinearly with the increase of the detecting amplitudes and bring nonlinear responses for the micro-shell resonator. Therefore, the experimental results in Fig. 2c show a bending-down curve away from the theoretical straight line. The amplitude responses of the micro-shell resonator between the input exciting voltages and the output detecting vibration are calculated and plotted in Fig. 2d, which is a parabola curve instead of a constant value, demonstrating that the larger vibrating amplitude brings greater actuation gain. The deviations from the linear value of the responses show an excellent linear fitting (*R*^2^ = 0.999782) with the square of the normalized amplitudes in Fig. 2e. The slope of the fitting line is close to the theoretical value of 3/4 in Eq. (6), which verifies the accuracy of the model of gain nonlinearity in electrostatic actuation.

### Influences of actuation gain nonlinearity on whole-angle mode

**Fig. 3:**
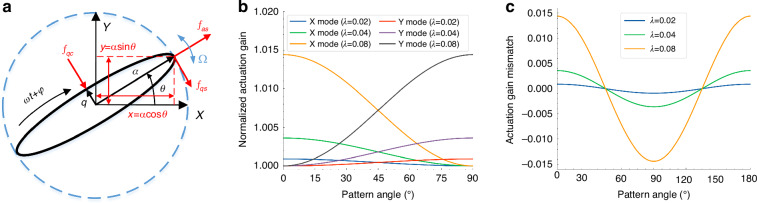
The actuation gain nonlinearity of whole-angle MSRG. **a** Elliptical orbit of the motion of MSRG. **b** Simulation results of the actuation gain at each pattern angle under different vibrating amplitudes. **c** Simulation results of the actuation gain mismatch by subtraction

For MSRG working in the whole-angle mode, the vibrating pattern can process freely to an arbitrary position, causing the amplitudes of two *n* = 2 modes to change during rotation. As a result, the degree of nonlinearity and the actual actuation gain will differ during rotation, which is a more common situation compared with MSRG working at a fixed location under FTR mode. The motion of MSRG working in the whole-angle mode is an elliptical orbit with a major axis of *a*, a minor axis of *q*, and a pattern angle of *θ*, as shown in Fig. 3a. Considering the phase-locking, energy-maintaining, and quadrature-nulling for MSRG working under whole-angle mode^30^, the vibrating displacements and the applied forces from electrostatic actuation of two *n* = 2 modes can be described as

8 $$\left\{\begin{array}{c}x=a\,\cos \theta \,\cos \omega t\\ y=a\,\sin \theta \,\cos \omega t\end{array},\left\{\begin{array}{c}|{F}_{x}|={k}_{dx}\left[1+3{\left(\frac{y}{{d}_{x0}}\right)}^{2}\right]\left({V}_{xc}\,\cos \omega t+{V}_{xs}\,\sin \omega t\right)\\ |{F}_{y}|={k}_{dy}\left[1+3{\left(\frac{y}{{d}_{y0}}\right)}^{2}\right]\left({V}_{yc}\,\cos \omega t+{V}_{ys}\,\sin \omega t\right)\end{array}\right.\right.$$

where

$$\left[\begin{array}{c}{V}_{xc}\\ {V}_{yc}\end{array}\right]=\left[\begin{array}{cc}\cos \theta & -\,\sin \theta \\ \sin \theta & \cos \theta \end{array}\right]\left[\begin{array}{c}{V}_{ac}\\ {V}_{qc}\end{array}\right],\left[\begin{array}{c}{V}_{xs}\\ {V}_{ys}\end{array}\right]=\left[\begin{array}{cc}\cos \theta & -\,\sin \theta \\ \sin \theta & \cos \theta \end{array}\right]\left[\begin{array}{c}{V}_{as}\\ {V}_{qs}\end{array}\right]$$

*V*_*ac*_, *V*_*as*_, *V*_*qc*_, and *V*_*qs*_ represent the output signals of control loops of frequency, energy, quadrature, and pattern angle, respectively. Substituting the displacements into the equations of the applied forces, the effective parts can be obtained as

9 $$\left\{\begin{array}{c}{f}_{x}={k}_{d}\left[1+\frac{9}{4}{\lambda }^{2}{\cos }^{2}\theta \right]{V}_{xc}\,\cos \omega t+{k}_{d}\left[1+\frac{3}{4}{\lambda }^{2}{\cos }^{2}\theta \right]{V}_{xs}\,\sin \omega t\\ {f}_{y}={k}_{d}\left[1+\frac{9}{4}{\lambda }^{2}{\sin }^{2}\theta \right]{V}_{yc}\,\cos \omega t+{k}_{d}\left[1+\frac{3}{4}{\lambda }^{2}{\sin }^{2}\theta \right]{V}_{ys}\,\sin \omega t\end{array}\right.$$

where *λ* = *a*/*d*_0_ represents the normalized amplitude ignoring the tiny distinction between capacitance gaps of two modes and *k*_*d*_ is the linear actuation gain after the compensations of the electrode errors^33,34^. According to Eqs. (8)–(9), when the vibrating pattern of MSRG precesses with the input rotation or by virtual-rotating force, the amplitudes of two modes change simultaneously, bringing their variations of the nonlinear strength and the actuation gain. The numerical simulation results for the actual actuation gain of two modes at each position with different normalized amplitudes are shown in Fig. 3b. At the pattern angle of 0°, the amplitude of X mode reaches a maximum with the largest actuation gain while the amplitude of Y mode is almost zero with an actuation gain near the unit. The case at the pattern angle of 90° is just the opposite. Therefore, such nonlinear effects aggravate the actuation gain mismatch between the two *n* = 2 modes, which is a function of cos2*θ* and grows quadratically with the increment of the vibrating amplitudes, as shown in Fig. 3c, bringing unavoidable influences on the whole-angle gyroscopes operating at large vibrating amplitude.

Combining Eqs. (8)–(9), the actual components in phase and quadrature *f*_*ac*_, *f*_*as*_, *f*_*qc*_, and *f*_*qs*_ of the forces *f*_*a*_, *f*_*q*_ along the major-axis and minor-axis of the ellipse orbit of MSRG can be expressed by the applied voltages of *V*_*ac*_, *V*_*as*_, *V*_*qc*_, and *V*_*qs*_ as

10 $$\begin{array}{rcl} \left[\begin{array}{c}{f}_{as}\\ {f}_{qs}\end{array}\right] &=& \left[\begin{array}{cc} 1+\frac{9}{16}{\lambda }^{2}+\frac{3}{16}{\lambda }^{2}\,\cos 4\theta & -\frac{3}{16}{\lambda }^{2}\,\sin 4\theta \\ -\frac{3}{16}{\lambda }^{2}\,\sin 4\theta & 1+\frac{3}{16}{\lambda }^{2}-\frac{3}{16}{\lambda }^{2}\,\cos 4\theta \end{array}\right] \left[\begin{array}{c}{V}_{as}\\{V}_{qs}\end{array}\right]\\ {\left[\begin{array}{c} {f}_{ac}\\ {f}_{qc}\end{array}\right]} &=&\left[\begin{array}{cc} 1+\frac{27}{16}{\lambda }^{2}+\frac{9}{16}{\lambda }^{2}\,\cos 4\theta & -\frac{9}{16}{\lambda }^{2}\,\sin 4\theta \\ -\frac{9}{16}{\lambda }^{2}\,\sin 4\theta & 1+\frac{9}{16}{\lambda }^{2}-\frac{9}{16}{\lambda }^{2}\,\cos 4\theta \end{array}\right]\left[\begin{array}{c}{V}_{ac}\\ {V}_{qc}\end{array}\right]\end{array}$$

Therefore, the dynamic equations of the control variables based on Lynch’s theory can be rewritten as

11 $$\begin{array}{c}\dot{E}=-\left[\frac{2}{\tau }+\Delta \left(\frac{1}{\tau }\right)\cos 2\left(\theta -{\theta }_{\tau }\right)\right]E-\frac{\sqrt{E}}{\omega }{k}_{d}\left[\left(1+\frac{9}{16}{\lambda }^{2}+\frac{3}{16}{\lambda }^{2}\,\cos 4\theta \right){V}_{as}-\frac{3}{16}{\lambda }^{2}\,\sin 4\theta {V}_{qs}\right]\\ \dot{\theta }=-\kappa \Omega +\frac{1}{2}\Delta \left(\frac{1}{\tau }\right)\sin 2\left(\theta -{\theta }_{\tau }\right)-\frac{{k}_{d}}{2\omega \sqrt{E}}\left[\left(1+\frac{3}{16}{\lambda }^{2}-\frac{3}{16}{\lambda }^{2}\,\cos 4\theta \right){V}_{qs}-\frac{3}{16}{\lambda }^{2}\,\sin 4\theta {V}_{as}\right]\\ \dot{Q}=-\frac{2}{\tau }Q-\Delta \omega \,\sin 2\left(\theta -{\theta }_{\omega }\right)E+\frac{\sqrt{E}}{\omega }{k}_{d}\left[\left(1+\frac{9}{16}{\lambda }^{2}-\frac{9}{16}{\lambda }^{2}\,\cos 4\theta \right){V}_{qc}-\frac{9}{16}{\lambda }^{2}\,\sin 4\theta {V}_{ac}\right]\\ \delta \dot{\varphi }=\dot{\varphi }^{\prime} +\frac{1}{2}\Delta \omega \,\cos 2\left(\theta -{\theta }_{\omega }\right)+\frac{{k}_{d}}{2\omega \sqrt{E}}\left[\left(1+\frac{27}{16}{\lambda }^{2}+\frac{9}{16}{\lambda }^{2}\,\cos 4\theta \right){V}_{ac}-\frac{9}{16}{\lambda }^{2}\,\sin 4\theta {V}_{qc}\right]\end{array}$$

where *E*, *Q*, and *δφ* represent the energy, quadrature error, and phase difference respectively, Δ(1/*τ*) and Δ*ω* are the damping anisotropy and frequency mismatch, *θ*_*τ*_ and *θ*_*ω*_ are the orientations of the principle axes of damping and frequency, Ω is the input rotation rate and *κ* is the angular gain. According to Eqs. (10)–(11), the gains of output voltages in four control loops all show the part of cos4*θ* and there are also force-couplings as the form of sin4*θ* between the loops of energy and angle, as well as the loops of quadrature error and phase difference. The unevenness of actuation gains and the coupling strength between control loops are proportional to the square of the normalized amplitudes. Under the normal working conditions of whole angle mode, the virtual rotation voltage of *V*_*qs*_ is absent and the output rate of the device with the extra angle-dependent bias resulting from the actuation gain nonlinearity can be expressed as

12 $$\dot{\theta }=-\kappa \varOmega +\frac{1}{2}\Delta (\frac{1}{\tau })\sin 2(\theta -{\theta }_{\tau })+(-\frac{3}{16\tau }{\lambda }^{2})\sin 4\theta$$

The extra part of sin4*θ* in bias comes from the control-force coupling of energy-maintaining loop, which is proportional to the damping of the resonator. Therefore, the actuation gain nonlinearity brings an extra 4th component to the angle-dependent bias, apart from the 2nd component caused by the damping anisotropy. And the influences of the actuation gain nonlinearity on the control loops of quadrature-nulling and phase-locking can be neglected due to a zero value of the voltage *V*_*ac*_ under whole-angle operation.

**Fig. 4:**
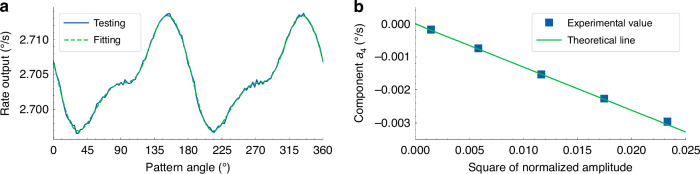
The infulence of actuation gain nonlinearity on rate output. **a** Testing and fitting results of the rate output. **b** The 4th component in bias versus the square of the normalized amplitudes

The experiments are carried out on MSRG working on the whole-angle mode to verify the theoretical analysis above. The practical normalized amplitudes in each group can be obtained by the method proposed in the next chapter. The rate outputs of the device are sampled continuously under a rotation rate of 5°/s on a turntable and then fitted by Fourier series of (*a*_2_sin2(*θ*-*θ*_2_)+*a*_4_sin4(*θ*-*θ*_4_)+*c*). For example, the testing and fitting results with a normalized amplitude of 0.152 are shown in Fig. 4a. Compared with the conventional linear model of whole-angle gyroscopes with only 2nd components of *a*_2_ of angle-dependent bias, the actuation gain nonlinearity brings extra 4th components of *a*_4_ to the bias, which causes the degeneration of output accuracy. The fitting results of the 4th components in rates with different normalized amplitudes are plotted in Fig. 4b. They are fitting linearly well (*R*^2^ = 0.999485) with the square of the normalized amplitudes, which also shows a good match with the linear model in Eq. (12) under a decay time of 81.7 s, verifying the model accuracy of the influences of actuation gain nonlinearity on whole-angle mode. The theoretical analysis and experimental results above explain that the actuation gain nonlinearity is the source of the remaining 4th component in angle-dependent bias after the calibration of capacitive detection nonlinearity^12^. In summary, the actuation gain nonlinearity will introduce an extra 4^th^ component into the angle-dependent bias, which is proportional to the square of the normalized amplitudes and inversely proportional to the decay time of the micro-shell resonator, causing negative impact on the output accuracy. Therefore, the performance of MSRG can be further improved by correction of the nonlinearity.

### Correction of actuation gain nonlinearity under whole-angle mode

As described in Eq. (9), the actuation gain nonlinearity introduces the over gain for the applied voltages of control force of *f*_*x*_ and *f*_*y*_, which relates to the pattern angle and normalized amplitude of MSRG. To realize the correction of actuation gain nonlinearity, the applied voltages in Eq. (9) should be modified by dividing the specific linearization coefficients before modulation, which can be expressed as

13 $$\left\{\begin{array}{c}{\tilde{V}}_{xc}=\frac{{V}_{xc}}{1+3\eta {\cos }^{2}\theta },{\tilde{V}}_{xs}=\frac{{V}_{xs}}{1+\eta {\cos }^{2}\theta }\\ {\tilde{V}}_{yc}=\frac{{V}_{yc}}{1+3\eta {\sin }^{2}\theta },{\tilde{V}}_{ys}=\frac{{V}_{ys}}{1+\eta {\sin }^{2}\theta }\end{array},\eta =\frac{3}{4}{\lambda }^{2}\right.$$

**Fig. 5:**
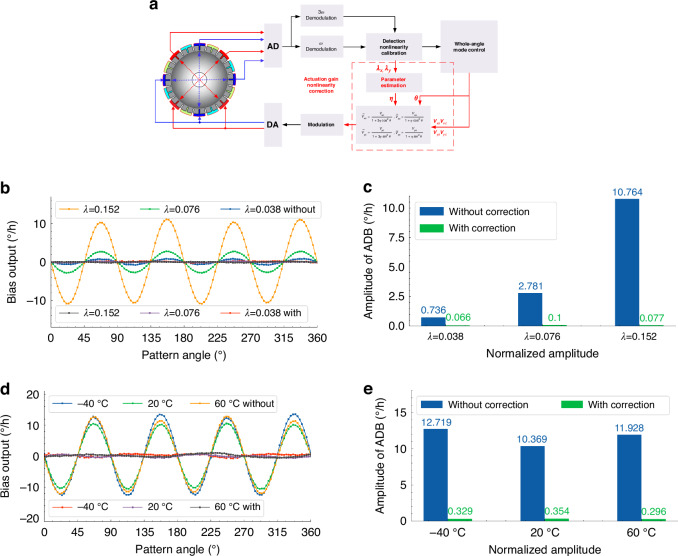
The correction of actuation gain nonlinearity for MSRG. **a** The implementation scheme of the actuation gain nonlinearity correction. (**b**), (**c**) The angle-dependent bias of MSRG without and with the correction of the actuation gain nonlinearity under different vibrating amplitudes. (**d**), (**e**) The angle-dependent bias of MSRG without and with the correction at three different temperatures

The parameter *η* refers to the normalized amplitude *λ*, which can be calculated as *λ*^2^ = *λ*_*x*_^2^ + *λ*_*y*_^2^, where *λ*_*x*_ and *λ*_*y*_ represent the normalized amplitudes of two *n* = 2 modes obtained during the process of calibration of capacitive displacement detection nonlinearity according to the harmonic-component relationship in Eq. (7). Therefore, the implementation of the correction method includes two parts as a parameter calculation part for online estimation of *η* and an output voltage modification part for gain correction of *V*_*xc*_, *V*_*xs*_, *V*_*xc*_, and *V*_*xs*_. The implementation scheme of the correction of the actuation gain nonlinearity is shown in Fig. 5a, which includes the estimation of the parameter *η* and the modification of the applying voltages. Under the compensation of damping anisotropy^35^, the angle-dependent bias of MSRG operating at different vibrating amplitudes is measured without and with the correction of the actuation gain nonlinearity by recording the rate output of the device at different pattern angles in the static state. The sampling time of each pattern angle is 120 s with an angle interval of 3° under a sampling frequency of 1 Hz, and the recording results are averaged as the bias output at each angle position. As shown in Fig. 5b, without the correction of the actuation gain nonlinearity, the angle-dependent bias shows a function of -sin4*θ* with large amplitudes. While with the correction, the angle-dependent bias mainly has the 2^nd^ component from the residual damping anisotropy with a great reduction of the 4th component. The amplitudes of 4th component in angle-dependent bias without and with the correction under different normalized amplitudes are plotted in Fig. 5c, which shows declines of over 90% of bias in each condition. The remaining 4^th^ components in angle-dependent bias under the correction of the actuation gain nonlinearity are all less than ± 0.0001°/s, which are close to the noise level of rate output of MSRG. Therefore, the extra angle-dependent bias resulting from the actuation gain nonlinearity can be considered to have been eliminated, proving the effectiveness of the correction method of the actuation gain nonlinearity.

In order to verify the environmental suitability of the real-time correction method, the angle-dependent bias of MSRG without and with the correction of the actuation gain nonlinearity are measured at three different temperatures of −40 °C, 20 °C and 60 °C. As shown in Fig. 5d, e, without the correction, the 4^th^ components in angle-dependent bias at these temperatures have the amplitude of 12.72°/h, 10.37°/h and 11.93°/h, respectively. According to Eq. (12), the extra bias caused by the actuation gain nonlinearity varies at different temperatures with the changes of decay times of the micro-shell resonator and capacitance gaps of electrostatic actuator, which cannot be directly removed by an open-loop compensation on bias. However, owing to the real-time correction of actuation gain nonlinearity, the 4^th^ component in angle-dependent bias can be reduced over 95% to less than ±0.0001°/s, achieving the same level as those at room temperature. The values of parameter *η* obtained from the real-time parameter estimation are 0.021169, 0.017804, and 0.015398 at three different temperatures, whose difference mainly comes from the changes of the capacitance gap with the temperature variation. Therefore, such a real-time correction method can realize both the suppression of actuation gain nonlinearity and the reduction of the angle-dependent bias under a wide temperature range.

Furthermore, electrostatic actuation using parallel-plate capacitor is also widely adopted in many other MEMS resonator devices, like disk resonator gyroscope^5^, cylindrical resonator gyroscope^6^, quad-mass gyroscope^7^, ring resonator gyroscope^8^, and so on. According to their design, they have the same model of electrostatic actuation and driving gain nonlinearity as shown in Eqs. (8)–(9). Therefore, the characteristics of model consistency of gain nonlinearity in electrostatic actuation make such correction method applicable to these MEMS resonator devices.

### Gyroscope performance characterization

**Fig. 6:**
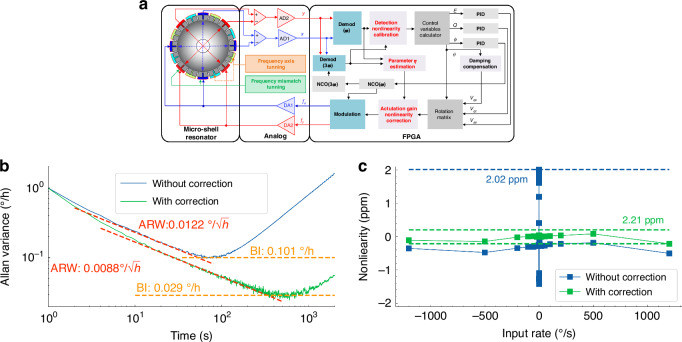
The performance of MSRG without and with the correction of the actuation gain nonlinearity. **a** Complete control system diagram of MSRG working on the whole-angle mode. **b** Allan variances of MSRG without and with the correction of the actuation gain nonlinearity. **c** Rate tests of MSRG with and without the correction of the actuation gain nonlinearity

The complete control system of MSRG working under the whole-angle mode consists of the resonator, analog front end, and digital controlled circuit, as illustrated in Fig. 6a. The analog front end includes C/V transformers, AD/DA converters, and stiffness-tuning-voltage generators. The vibrating displacements of the resonator are converted by the C/V transformer into voltage and digitized by high-resolution ADCs. The whole-angle control mode is implemented in the FPGA platform, which also contains the module of damping anisotropy compensation^35^, capacitive displacement detection calibration and actuation gain nonlinearity correction.

The MSRG without and with the correction of the actuation gain nonlinearity is tested under room temperature for 8 h with the sampling rate of 1 Hz. With the correction, the drift of the rate output is reduced during static test due to the suppression of the angle-dependent bias. Therefore, the BI of MSRG is improved from 0.101°/h to 0.029°/h, with a slight promotion of ARW from 0.0122°/√h to 0.0088°/√h, as illustrated in Fig. 6b. Besides, the rate test is performed on MSRG without and with the correction at 27 rate points ranging between ± 1200°/s. For each rotation point, the rate of MSRG is acquired for 100 s under the sampling rate of 1 Hz. The measurement range of MSRG can reach ±1200°/s with a scale factor of about 0.54073348. As shown in Fig. 6c, the line fitting residuals of the rate test of MSRG without the correction are larger at low-speed rotation (< 2°/s) due to extra bias caused by the actuation gain nonlinearity. While with the correction, the scale-factor nonlinearity (SFN) of MSRG is improved by about one order of magnitude from 2.02 ppm to 0.21 ppm. Therefore, the correction method can remove the negative influences from strong nonlinear effects on MSRG operating at a large vibrating amplitude for a better SNR and improve both the static and dynamic performance of the gyroscope.

In summary, the correction of the actuation gain nonlinearity can improve the BI, ARW, and SFN performance of whole-angle MSRG especially when operating at a large vibrating amplitude for a lower noise level. With the correction, the trade-off between larger bias and better noise with increment of the vibrating amplitude is removed, making MSRG achieve both better BI and SNR performance.

The performances of whole-angle MEMS gyroscopes published in recent literatures^36–40^ are provided in Table 1 for comparison. The data evidently indicates that whole-angle MSRG in this work achieve an improvement from our previous work^12^ and exhibits advanced performance compared with other whole-angle MEMS gyroscopes. Therefore, MSRG with such outstanding performance will have wide applications in aerospace segment^41,42^, robotics^43^, drone navigation^44,45^, gyrocompass^46,47^ and automobile^48^. The performance improvement of the gyroscope can enhance the accuracy of inertial navigation, attitude measurement and north finding.

**Table 1:** Performance Summary of Whole-Angle MEMS Gyroscopes

Work	Type	BI (°/h)	ARW (°/√h)	SF (ppm)
[36]	Ring-Shape gyroscope	1.1	0.18	N/A
[37]	Quad Mass gyroscope	N/A	N/A	7
[38]	Donut-Mass gyroscope	0.1	N/A	N/A
[39]	Dual Foucault Pendulum gyroscope	0.2	N/A	10
[40]	Micro-Shell Resonator gyroscope	0.579	N/A	N/A
[12]	Micro-Shell Resonator gyroscope	0.0673	N/A	0.79
This work	Micro-Shell Resonator gyroscope	0.029	0.0088	0.21

## Conclusion

In this paper, the influence mechanism and suppression approach of the gain nonlinearity in electrostatic actuation on the whole-angle micro-shell resonator gyroscope is studied. The model of the actuation gain nonlinearity is established and the dynamical control equations of the whole-angle mode are derived in the nonlinear regime. The theoretical analysis demonstrates that the actuation gain nonlinearity causes control-force coupling and brings extra angle-dependent bias with the 4^th^ component for the whole-angle gyroscopes, which is proved by the experimental results fitting well with the numerical simulation. Furthermore, a real-time correction method is proposed to restore a linear response of the electrostatic actuation, which is realized by the gain modification with the online estimation of specific linearization coefficients based on the harmonic-component relationship in capacitive detection. The effectiveness of such real-time correction is verified by the suppression of the 4th component in angle-dependent bias from about 0.003°/s to less than 0.0001°/s at different temperatures. With the correction of actuation gain nonlinearity for whole-angle MSRG, the performance of BI is improved by about 3.5 times from 0.101°/h to 0.029°/h with an ARW of 0.0088°/√h and the scale factor nonlinearity (SFN) is reduced by almost one order of magnitude from 2.02 ppm to 0.21 ppm. Therefore, MSRG can still achieve both better BI and SFN performances even when operating at large amplitude for a better SNR, benefiting from the elimination of angle-dependent bias by the correction of the actuation gain nonlinearity. More importantly, such a correction method can be used on other resonators with parallel-plate capacitive actuators for a linear electrostatic actuation even under large vibrating amplitude.
